# Diagnostic value of artificial intelligence-assisted endoscopy for chronic atrophic gastritis: a systematic review and meta-analysis

**DOI:** 10.3389/fmed.2023.1134980

**Published:** 2023-05-02

**Authors:** Yanting Shi, Ning Wei, Kunhong Wang, Tao Tao, Feng Yu, Bing Lv

**Affiliations:** ^1^Department of Gastroenterology, Zibo Central Hospital, Zibo, Shandong, China; ^2^School of Computer Science and Technology, Shandong University of Technology, Zibo, Shandong, China

**Keywords:** chronic atrophic gastritis, artificial intelligence, deep learning, endoscopy, systemic review, meta-analysis

## Abstract

**Background and aims:**

The diagnosis of chronic atrophic gastritis (CAG) under normal white-light endoscopy depends on the endoscopist's experience and is not ideal. Artificial intelligence (AI) is increasingly used to diagnose diseases with good results. This review aimed to evaluate the accuracy of AI-assisted diagnosis of CAG through a meta-analysis.

**Methods:**

We conducted a comprehensive literature search of four databases: PubMed, Embase, Web of Science, and the Cochrane Library. Studies published by November 21, 2022, on AI diagnosis CAG with endoscopic images or videos were included. We assessed the diagnostic performance of AI using meta-analysis, explored the sources of heterogeneity through subgroup analysis and meta-regression, and compared the accuracy of AI and endoscopists in diagnosing CAG.

**Results:**

Eight studies that included a total of 25,216 patients of interest, 84,678 image training set images, and 10,937 test set images/videos were included. The results of the meta-analysis showed that the sensitivity of AI in identifying CAG was 94% (95% confidence interval [CI]: 0.88–0.97, I^2^ = 96.2%), the specificity was 96% (95% CI: 0.88–0.98, I^2^ = 98.04%), and the area under the summary receiver operating characteristic curve was 0.98 (95% CI: 0.96–0.99). The accuracy of AI in diagnosing CAG was significantly higher than that of endoscopists.

**Conclusions:**

AI-assisted diagnosis of CAG in endoscopy has high accuracy and clinical diagnostic value.

**Systematic review registration:**

http://www.crd.york.ac.uk/PROSPERO/, identifier: CRD42023391853.

## 1. Introduction

According to global cancer data released by the International Agency for Research on Cancer (IARC), approximately 1.09 million new cases of gastric cancer (GC) and approximately 770,000 deaths were recorded in 2020, ranking fifth in incidence and fourth in mortality (1). Professor Correa proposed that the development of intestinal-type gastric adenocarcinoma follows a cascade pattern: from normal gastric mucosa to chronic non-atrophic gastritis (CNAG), followed by chronic atrophic gastritis (CAG), atypical hyperplasia (dysplasia), and finally to intestinal GC (2, 3). This model has been widely recognized (4–6). A Dutch study found that the annual incidence of GC was 0.1 and 0.25% for patients with atrophic gastritis (AG) and intestinal metaplasia (IM), respectively (7). The risk of GC is higher in the CAG population in East Asia, and a long-term follow-up study in Japan found that the 10-year cumulative GC incidence after *Helicobacter pylori* eradication ranged from 3.4 to 16% in patients with moderate-to-severe atrophy and from 11 to 16% in patients with IM (8). A Korean study found that 52.5% of patients with diffuse GC had AG, and 18.4% had severe AG (9). CAG is a precancerous disease; therefore, early diagnosis of CAG is vital in preventing GC (10, 11).

Gastroscopic biopsy of the gastric mucosal tissue for histopathological analysis is the “gold standard” for diagnosing CAG (12, 13). In clinical endoscopy, gastric mucosal tissues are first observed by conventional white-light endoscopy, and the need for endoscopic biopsy depends on the endoscopist's experience. A study showed that the sensitivity of conventional normal white light endoscopy (WLE) for diagnosing CAG is only 42% (14). Another study showed that the diagnostic sensitivity and specificity of conventional WLE for gastric mucosal atrophy were 61.5 and 57.7% for the gastric sinus, and 46.8 and 76.4% for the gastric body, respectively (15). In recent years, electronic or virtual color endoscopy has received increasing attention for the diagnosis CAG because it allows for more accurate detection of lesion (16, 17). However, these advanced techniques are usually further steps taken when CAG is already suspected after WLE, and the accuracy of endoscopic diagnosis still relies on the endoscopist's experience (18). It is difficult to avoid missed diagnoses due to fatigue and inexperience of endoscopists. Biopsies are expensive and time-consuming, and could increase the risk of gastric mucosal bleeding. Therefore, developing a method to identify CAG objectively, stably, and accurately is important to reduce the workload of endoscopists and to prevent the occurrence of GC.

**Figure 1:**
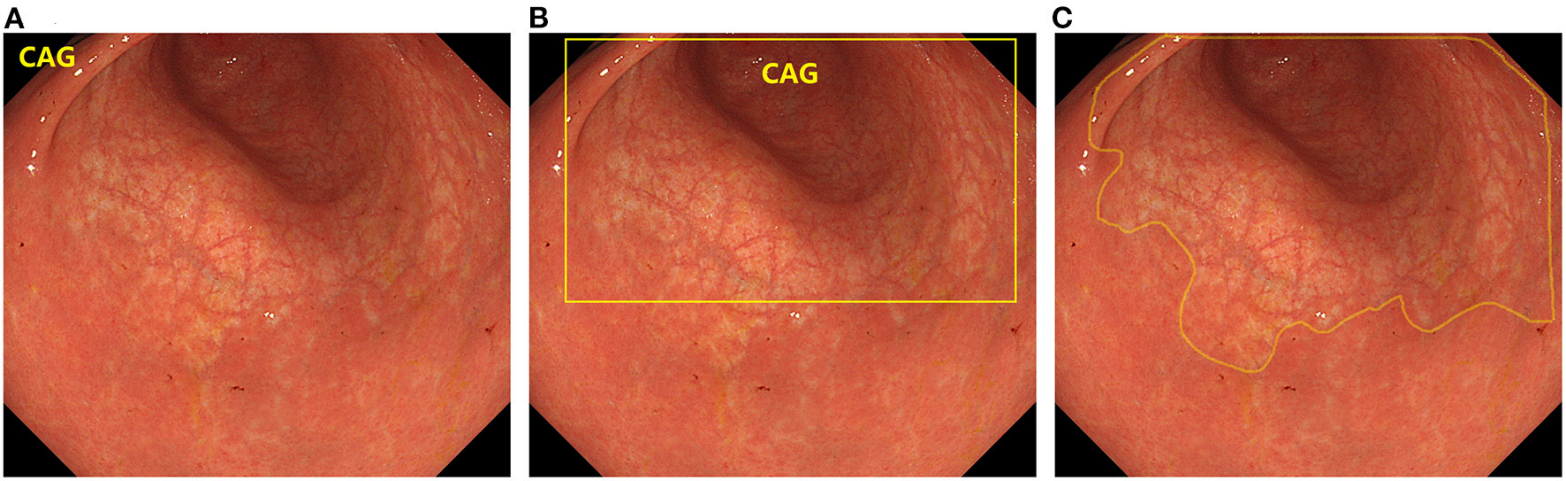
The difference between the three types of deep learning algorithms. **(A)** Image classification. **(B)** Object detection. **(C)** Semantic segmentation.

In recent years, artificial intelligence (AI) technology, particularly deep learning, has become a popular analytical tool for medical imaging (19). AI techniques have been widely used in computer-aided diagnosis (20–22). In computer vision, the primary tasks of deep learning include image classification, object detection, and semantic segmentation. Image classification determines the category to which a given image belongs, and typical algorithms include VGGNet (23), ResNet (24), TResNet (25), and SE-ResNet (26). Object Detection is used to identify objects and their positions in the image and frame them with rectangles, such as R-CNN (27), YOLO (28, 29), and SSD (30). Semantic segmentation involves recognizing the objects and their positions in the image and outlines them in the image. Typical algorithms are U-Net (31), UNet++ (32), and DeeplabV3 (33). The differences between the three algorithms are shown in Figure 1. If AI can accurately identify CAG on endoscopy, it will greatly alleviate the current problems faced in CAG diagnosis. However, this requires a combination of existing studies to quantify the accuracy of AI in detecting CAG.

This meta-analysis aimed to systematically review and analyze the diagnostic performance of AI in CAG. It mainly includes the overall performance of AI in CAG diagnosis, comparison between AI and endoscopists, and analysis of various factors that influence the diagnostic performance of AI.

## 2. Methods

This systematic review followed the guidelines of the preferred reporting items for systematic review and meta-analysis of diagnostic test accuracy studies (PRISMA-DTA) (34). The PRISMA-DTA checklist is shown in Supplementary Table S1. Before initiating the study, it was registered with the International Prospective Register of Systematic Reviews (PROSPERO) on October 31, 2022 (ID: CRD42022371134). All data for this study were collected from the literature, and ethical approval was not required.

### 2.1. Searching strategy

We systematically searched the following four databases: PubMed, Embase, Web of Science, and Cochrane Library. PubMed, Embase, and Web of Science are widely used medical databases, while the Cochrane Library is a database related to evidence-based medicine. The last search was conducted on November 21, 2022 and covered all articles in the four databases up to the time of the search. The keywords searched included ten terms related to AI and five related to CAG. The keywords related to AI included “artificial intelligence,” “deep learning,” “machine learning,” “computer aided diagnosis,” “neural networks,” “transfer learning,” and “transformer.” The keywords related to CAG include “atrophic,” “atrophy,” “gastritis,” “intestinal metaplasia,” and “endoscopy.” The search strategy is presented in Supplementary Table S2.

### 2.2. Study selection

Two authors (NW and FY) independently screened the retrieved articles to determine whether they met the inclusion criteria. When judgment could not be made based on the title and abstract, the full text of the article was reviewed. All disagreements were resolved through discussion with YS.

The inclusion criteria were as follows: (1) Studies using AI to diagnose CAG; (2) diagnosis was based on endoscopic images or videos; (3) compositions of the dataset were described in detail; (4) clear diagnostic criteria, pathology or expert consensus.; (5) studies that provided the number of true positives (TP), false positives (FP), true negatives (TN), and false negatives (FN), either directly, or indirectly; and (6) for similar studies by the same author or team, preference was given to prospective studies and those with larger sample sizes.

The exclusion criteria were as follows: (1) studies without primary data (e.g., narrative reviews, comments, letters); (2) studies whose full text was unavailable; and (3) studies with insufficient data to obtain TP, FP, TN, and FN. We contacted the corresponding author of the article by email to obtain relevant data and will include the article if we can receive a response before the official publication of this meta-analysis.

### 2.3. Data extraction

NW and TT independently extracted data for inclusion in the study, and all disagreements were resolved by discussion with YS. For each study, the following data were collected: first author, year of publication, country or region, diagnostic criteria, endoscopy type, data source, number of patients, number of images/lesions in the training set, number of patients/images/videos/lesions in the test set, AI algorithm, and number of TP, FP, TN, and FN. In a study with multiple sets of test data, we selected according to the following rules: (1) preference for prospective test results; (2) preferences were given to external test results; and (3) preference was given to test results with a large sample size.

We also extracted data related to the endoscopic diagnosis of CAG by endoscopists to compare with the diagnostic performance of AI.

### 2.4. Quality assessment

The most commonly used instrument for evaluating the quality of diagnostic trials is the Quality Assessment of Diagnostic Accuracy Studies Version 2 (QUADAS-2) tool (35). There is no widely accepted assessment tool for the quality assessment of diagnostic assistance provided by AI. We referenced two studies (36, 37) and added four questions to QUADAS-2 to precisely assess the studies included in this meta-analysis. The following were also added to the patient selection section: (1) whether the data source and data set division is described in detail. (2) Whether the preprocessing process of the data was described. The following were added to the index test section: (1) whether the type of endoscopy used is clearly described and (2) whether the test set setting is reasonable.

### 2.5. Statistical analysis

To evaluate the performance of AI in diagnosing CAG, we summarized the sensitivity, specificity, positive likelihood ratio (PLR), negative likelihood ratio (NLR), diagnostic odds ratio (DOR), and 95% confidence intervals (CI) based on the extracted TP, TN, FP, and FN data. The summary receiver operating characteristic (sROC) curve was plotted and the area under the curve (AUC) was calculated. The higher the PLR value, the better the AI can confirm the diagnosis of CAG. The smaller the NLR value, the better the AI can exclude CAG. AUC and DOR are comprehensive indicators of diagnostic performance, and larger values indicate stronger diagnostic capability of AI.

Publication bias was analyzed using the Deek's test and funnel plot, and publication bias was significant at *P* < 0.05. To explore the accuracy of AI in identifying CAG in different subgroups and possible sources of heterogeneity in the study, we performed subgroup analysis and meta-regression with the following grouping conditions: training set size, AI algorithm type, endoscope type, test set as image or video, and diagnostic criteria. The heterogeneity of the included studies was tested using the Cochrane Q test, with I^2^ > 50% or a *P* value < 0.05, indicating significant heterogeneity.

We assessed the quality of the included studies using Review Manager 5.4 (Cochrane Collaboration, Oxford, UK) and completed all statistics and analyses using Stata/SE 16.0 (StataCorp LLC, College Station, TX, USA) with the MIDAS package installed.

## 3. Result

### 3.1. Included studies

**Figure 2:**
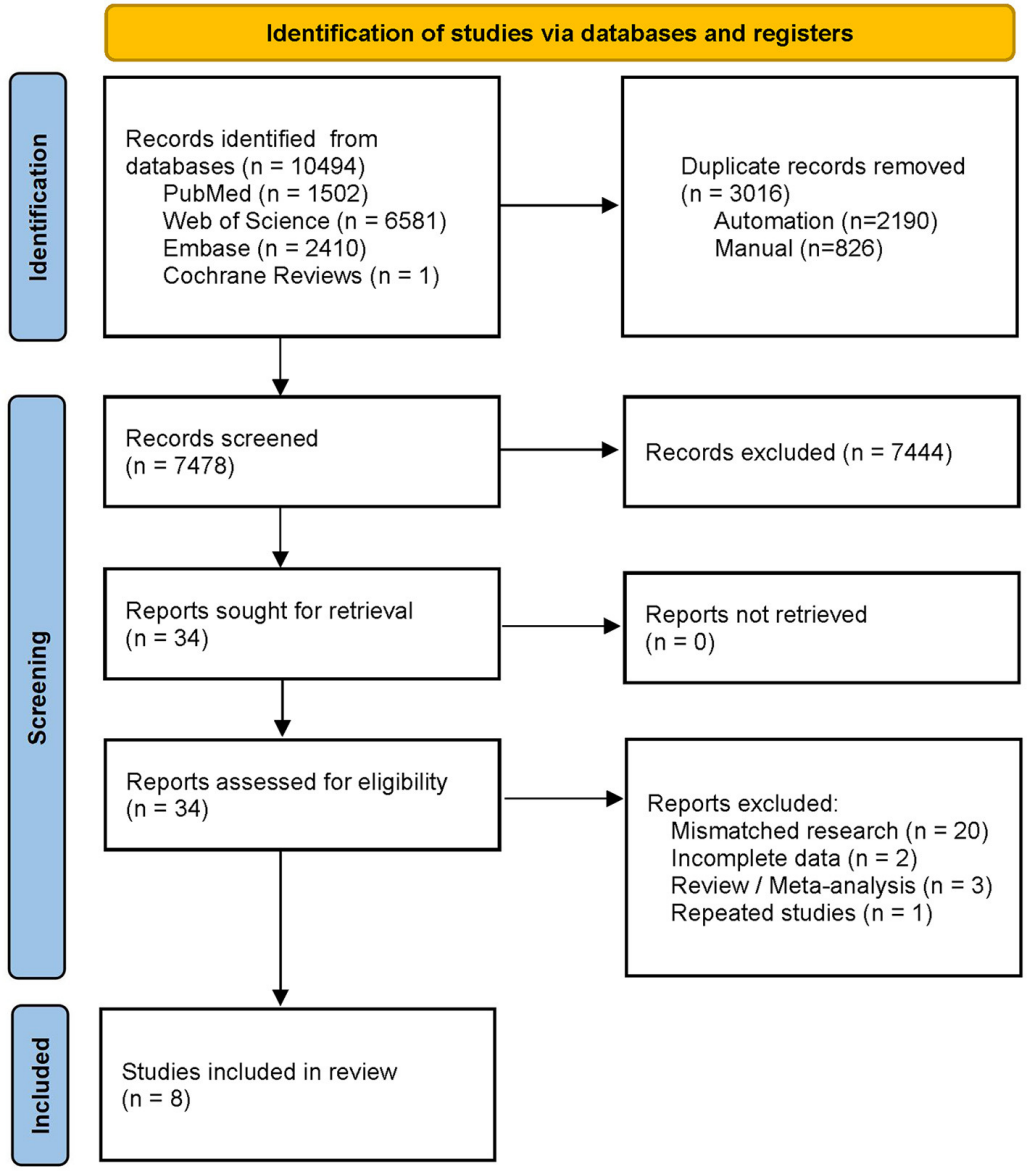
PRISMA flow diagram for study selection.

The literature search retrieved 10,494 studies, of which 3,016 duplicates were automatically removed using a software. A total of 7,444 mismatched studies were manually removed by reading the abstracts. After reading the full texts of the remaining articles, 26 more studies were excluded. Finally, eight studies were included in this meta-analysis (38–45); details of the articles are shown in Table 1, and the flow chart of study selection is shown in Figure 2. One study (46) was excluded because TP, TN, FP, and FN data were unavailable. Two studies (43, 47) were from the same team, one of which (47) constructed and tested a CAG diagnostic model, and the other (43) performed a further test; hence, we selected the more extensive test set of data (43) included in this meta-analysis. Two other studies (41, 48) were also from the same team; one study (41) used AI to identify AG and IM, and another study (48) added the identification of GC to the former, and we chose the first (41) to be included in this meta-analysis. Three studies were excluded because only IM was identified (49–51).

**Table 1:** Details of the included studies.

**Study**	**Country/region**	**Study center**	**Study design**	**Endoscopy**	**Algorithm**	**Standard reference**	**Patients(n)**	**Train set images(n)**	**Test set type**	**TP**	**FP**	**TN**	**FN**
Guimarães et al. (38)	Germany	Single	Retrospective	WLI	VGG16	Pathology	136	200	Internal image	30	5	0	35
Qu et al. (39)	China	Multi	Prospective	WLI/ Chromoendoscopy	YoloV3	Expert consensus	9,869	37,587	Prospective video	305	8	100	5,286
Mu et al. (40)	China	Multi	Retrospective	WLI	Unet++ and Resnet-50	Pathology	5,190	8,147	Retrospective video	41	3	1	35
Lin et al. (41)	China	Multi	Retrospective	WLI	TResNet	Pathology	2,741	6,309	External image	357	22	11	706
Luo et al. (42)	China	Multi	Retrospective	WLI	Resnet-50	Pathology	4,005	7,457	External image	87	15	13	85
Zhao et al. (43)	China	Single	Prospective	Unclear	U-NET	Pathology	1,711	3,703	Prospective video	284	10	54	328
Xu et al. (44)	China	Multi	Prospective	NBI BLI	VGG16	Pathology	934	4,138	Prospective video	111	17	5	63
Yang et al. (45)	China	Single	Retrospective	WLI LCI	SE-ResNet	Pathology	630	17,137	Internal image	1,393	45	67	1,415

WLI, White light imaging; LCI, Linked color imaging; NBI, Narrow-Band imaging; BLI, Blue laser imaging.

### 3.2. Quality assessment

**Figure 3:**
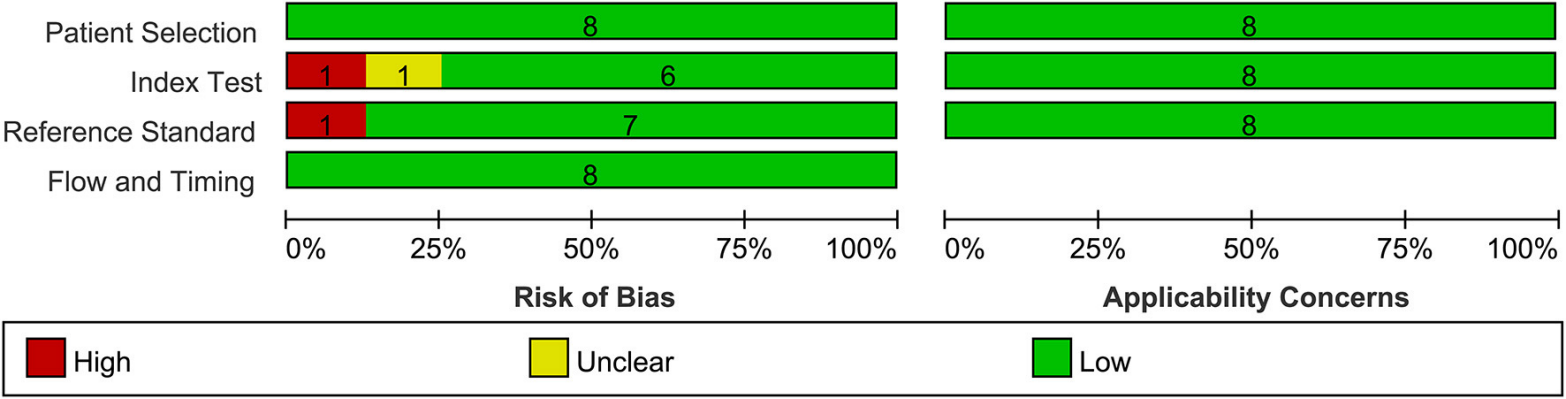
Summary of risk of bias and applicability of concerns graph.

The included studies were evaluated using the QUADAS-2 tool. Five articles had low levels of bias, two articles had a high bias, and one article had an unclear bias, as shown in Figure 3. Yang et al. (45) evaluated the model using a data-enhanced test set and considered it to have a high risk of bias. The study by Zhao et al. (43) did not mention the type of endoscope used and was considered to have an unclear risk of bias.

The study by Qu et al. (39) did not use pathological findings as a diagnostic criterion but used expert consensus. There is a discrepancy between CAG diagnosis through endoscopic images and pathological results. However, the use of chromoendoscopy images (52) and the consensus of experts can reduce these errors. After discussions among all the authors, we decided to include this study, but it had a high risk of bias.

### 3.3. Characteristics of the included studies

Five of the eight studies were retrospective (38, 40–42, 45), and three were prospective (29, 43, 44). All eight studies used deep-learning techniques: five used image-classification algorithms (38, 41, 42, 44, 45), one used an object-detection algorithm (39), one used a semantic-segmentation algorithm (43), and one used a combination of image classification and semantic segmentation (40). All studies were tested using static image models, and four studies used prospective videos to validate the models further (39, 40, 43, 44). Regarding the type of endoscopy, four studies included only normal white-light endoscopy (38, 40–42), three used enhanced endoscopy (39, 44, 45), and one did not specify the type of endoscopy (43). Seven studies used pathology as the gold standard, and one used expert consensus as the decision criterion (39).

Two studies used the internal image test set (38, 45), two studies used the external image test set (41, 42), one study used the retrospective video test set (40), and three studies used the prospective video test set (39, 43, 44). The number of test set images or videos equals the sum of TP, FP, TN, and FN values. The type and number of test sets mentioned here refer only to the data extracted by this systematic review.

Qu et al. (39) developed a complete gastrointestinal lesion identification system that can identify ten diseases, including CAG. Only test data for CAG identification were included in the meta-analysis.

Mu et al. (40) developed an AI-based assisted diagnosis system for identifying four lesions: GA, IM, erosive, and hemorrhagic gastritis. The entire system consisted of five deep-learning models. We extracted the results of the DCNN2 model to identify CAG.

Lin et al. (41) developed a computer-aided decision system for identifying the GA and IM. We obtained TP, FP, TN, and FN data by calculation, and the GA and IM identification results were combined and included in this meta-analysis.

Luo et al. (42) developed two AI models. Model 1 was used to identify CAG and the degree of atrophy, and both training and testing of model 1 used gastric sinus images. Model 2 was used to identify CAG, and the performance of model 2 was evaluated using three test sets, referred to as test sets 3, 4, and 5 in this study. Test set 3 was the internal test set, and test sets 4 and 5 were the external test sets; however, test set 5 did not contain the gastric sinus images. We selected the evaluation results of model 2 in test set 4 for inclusion in this meta-analysis.

Xu et al. (44) developed a real-time detection system for identifying GA and IM and tested it on internal, external, and prospective videos. We obtained TP, FP, TN, and FN data by calculating the recognition results of GA and IM and recomputed the sensitivity, specificity, and accuracy of CAG.

### 3.4. Performance of AI in CAG diagnosis

**Figure 4:**
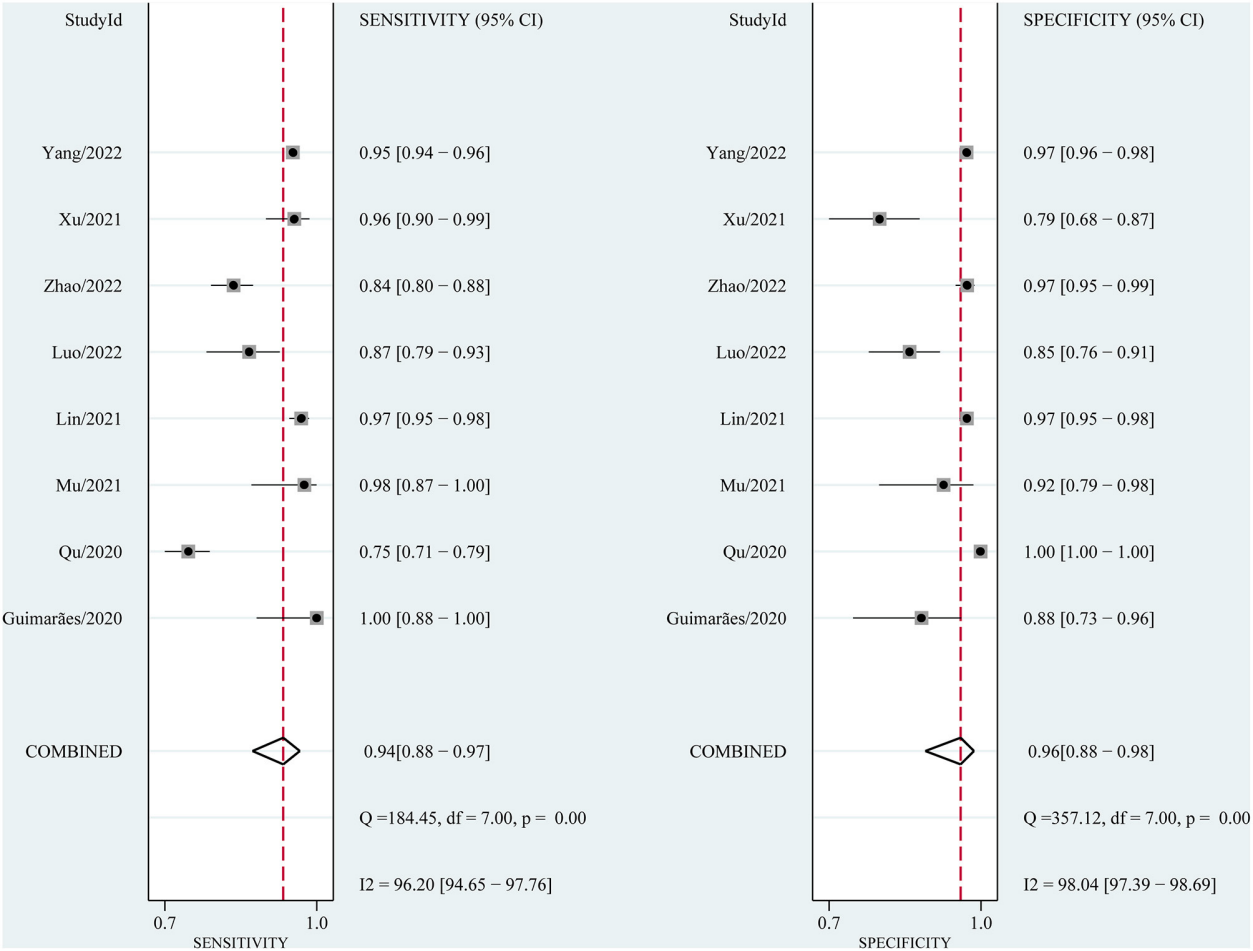
Forest plot of sensitivity and specificity of AI in identifying CAG.

We performed a pooled analysis of the eight included studies to assess the overall performance of AI in the endoscopic-assisted diagnosis of CAG. As shown in Figure 4, the pooled sensitivity and specificity were 94% (95% CI: 0.88–0.97, I^2^ = 96.2%) and 96% (95% CI: 0.88–0.98, I^2^ = 98.04%), respectively, both of which showed significant heterogeneity. The pooled PLR, NLR, and DOR were 21.58 (95% CI: 7.91–58.85, I^2^ = 96.23%), 0.07 (95% CI: 0.04–0.13, I^2^ = 95.95%, Supplementary Figure S1), and 320.19 (95% CI: 128.5–797.84, I^2^ = 100%, Supplementary Figure S2), respectively. The PLR greater than 10 indicated that AI could confirm the diagnosis of CAG. The NLR less than 0.1 indicated that AI could effectively exclude CAG. The DOR is significantly greater than 1, which indicates that AI has a good discrimination of CAG.

**Figure 5:**
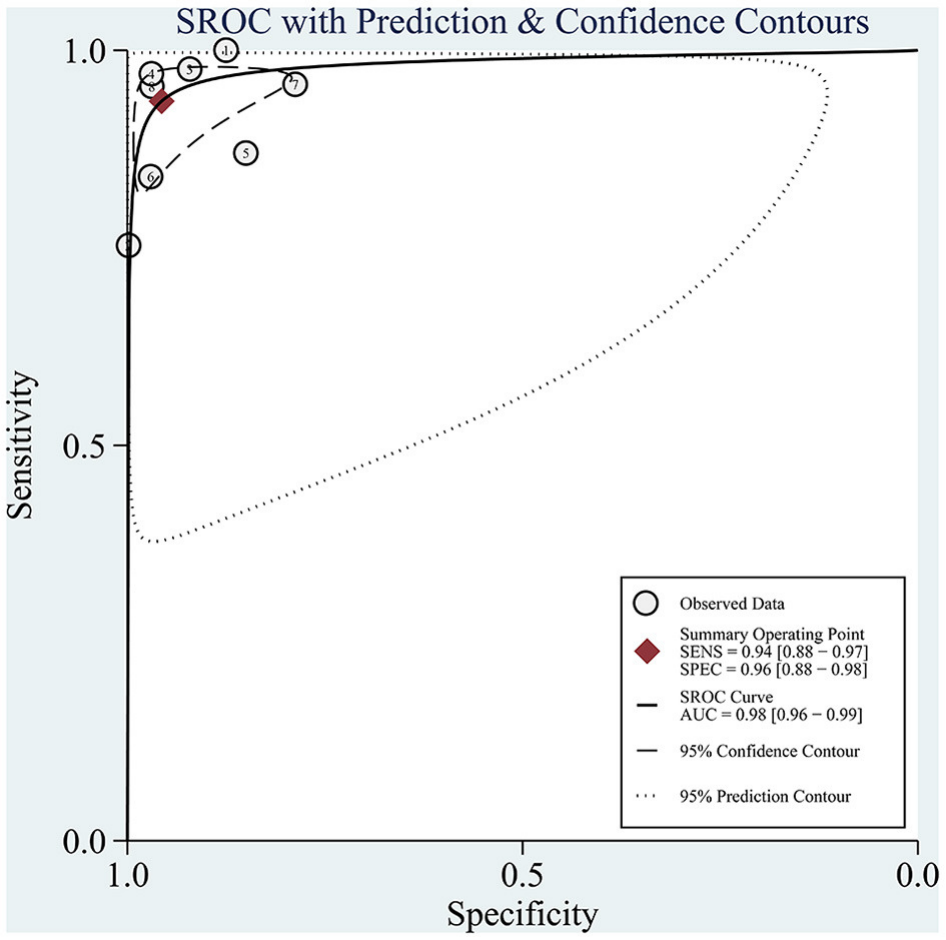
sROC curves for the diagnosis of CAG using AI. Each circle indicates an individual study, red diamond represents summary sensitivity and specificity.

The sROC curve is shown in Figure 5, and the AUC was 98% (95% CI: 0.96–0.99). The sROC is a composite index reflecting the sensitivity and specificity of continuous variables, and the closer the AUC value is to 1, the better the diagnosis. This shows that AI has excellent performance in the diagnosis of CAG.

**Figure 6:**
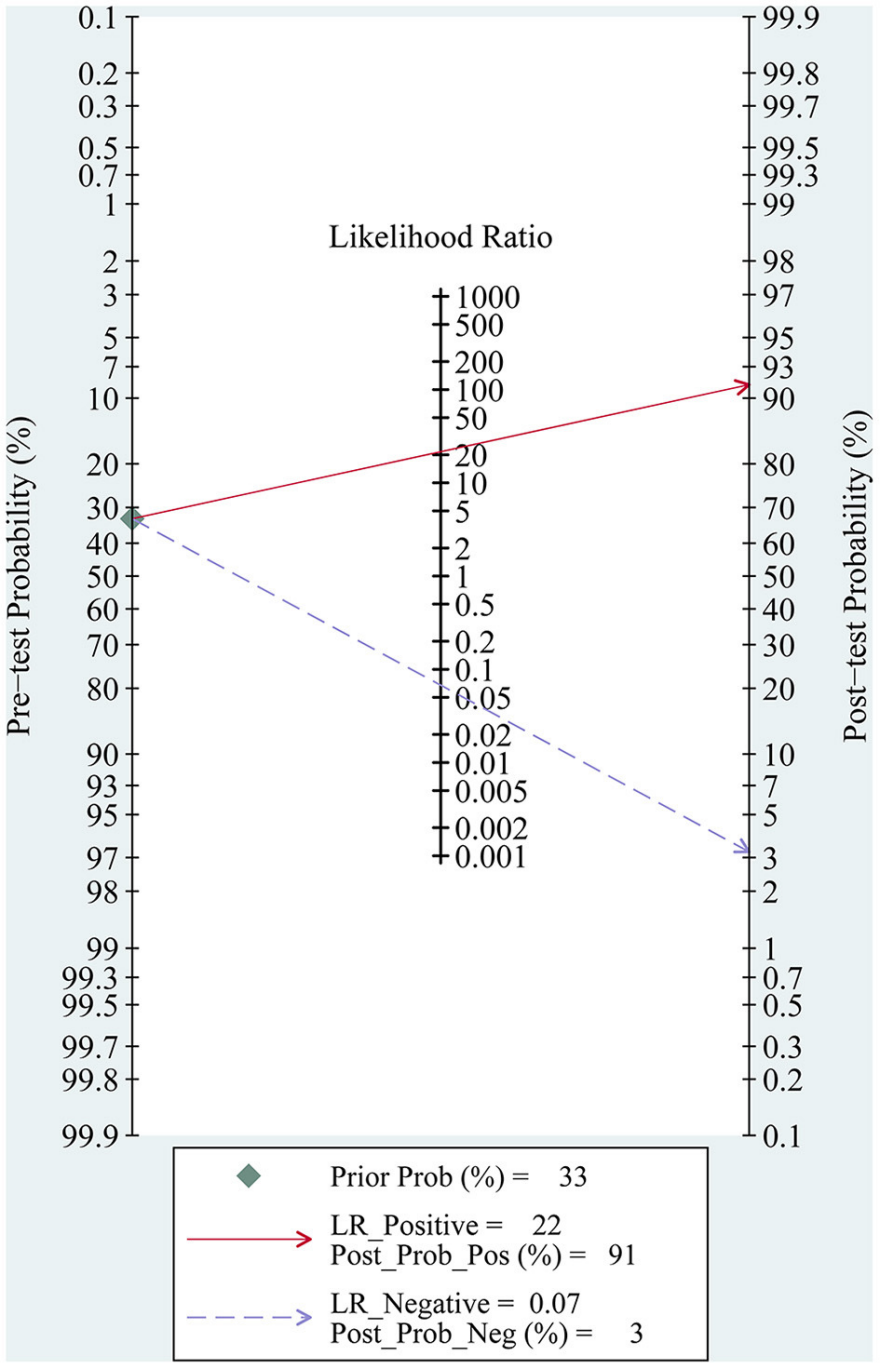
Fagan nomogram of the accuracy of AI in the diagnosis of CAG.

We evaluate the clinical diagnostic capability of the AI models by means of Fagan plot (Figure 6). The global prevalence of CAG in the general population when biopsy is considered to be 33% (95% CI: 0.26–0.41) (53). We set the pretest probability to 33%, with a 91% probability of a positive patient being diagnosed with CAG and a 3% probability of a negative patient being diagnosed with CAG. The above data indicate that the diagnosis of CAG with AI has good accuracy and important clinical application.

### 3.5. Subgroup analysis and meta-regression

**Figure 7:**
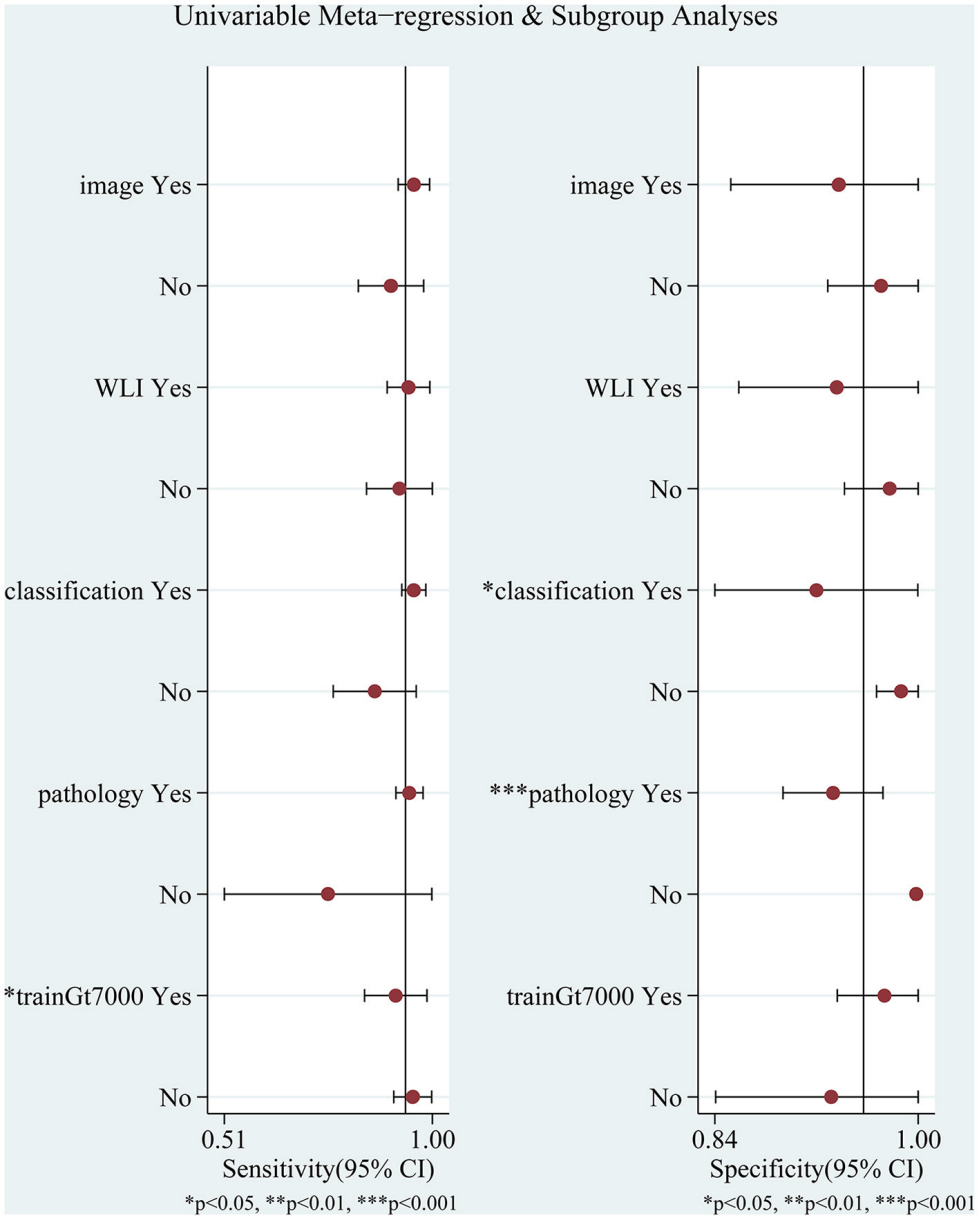
Meta-regression and subgroup analyses for potential sources of heterogeneity. WLI: endoscopic imaging type is WLI or other; image: test set as image or video; classification: AI algorithm an image classification algorithm or other algorithms; pathology: whether to use pathology as a diagnostic criterion; trainGt7000: whether the number of images was greater than 7,000.

The I^2^ values for pooled sensitivity and specificity were 96.2 and 98.04%, respectively, indicating high heterogeneity of the included studies. We performed a subgroup analysis and meta-regression analysis to explore possible sources of heterogeneity based on the test set type (image or video), endoscopic imaging type (WLI or other), algorithm type (image classification or object detection with semantic segmentation), diagnostic criteria (pathology or other), endoscope type (pure normal white light endoscopy or other), and the number of training set images (whether the number of images was greater than 7,000. In 7,000 is a median that exactly divides the eight studies equally into two groups). The results are shown in Figure 7 and Table 2.

**Table 2:** Subgroup analyses and meta-regression results.

**Parameter**	**Category**	**Studies(n)**	**Sensitivity (95%CI)**	***P***	**Specificity (95%CI)**	***P***
WLI	Yes	5	0.94 (0.89–0.99)	0.54	0.94 (0.86–1.00)	0.38
	No	3	0.92 (0.84–1.00)		0.98 (0.94–1.00)	
Image	Yes	4	0.96 (0.92–0.99)	0.63	0.94 (0.85–1.00)	0.55
	No	4	0.90 (0.82–0.98)		0.97 (0.93–1.00)	
Classification	Yes	5	0.96 (0.93–0.98)	0.72	0.92 (0.84–1.00)	0.03
	No	3	0.86 (0.77–0.96)		0.99 (0.97–1.00)	
Pathology	Yes	7	0.94 (0.91–0.98)	0.18	0.93 (0.89–0.97)	0.00
	No	1	0.75 (0.51–1.00)		1.00 (1.00–1.00)	
TrainGt7000	Yes	4	0.91 (0.84–0.99)	0.04	0.97 (0.94–1.00)	0.28
	No	4	0.95 (0.91–1.00)		0.93 (0.84–1.00)	

WLI, endoscopic imaging type is WLI or other; image, test set as image or video; classification, AI algorithm an image classification algorithm or other algorithms; pathology, whether to use pathology as a diagnostic criterion; trainGt7000, whether the number of images was greater than 7,000.

Meta-regression analysis showed that the number of training set images (*p* = 0.04) significantly affected sensitivity and could be a potential source of sensitivity heterogeneity. Algorithm type (*p* = 0.03) had a significant effect on specificity, and diagnostic criteria (*p* = 0.00) had a highly significant effect on specificity, which may be a potential source of heterogeneity in specificity. Other factors had no statistically significant effects on sensitivity and specificity. Although the study by Zhao et al. (43) did not specify the type of endoscopic imaging, we categorized it as a pure white light imaging or another form of imaging, neither of which had a significant effect on the results.

To further explore the heterogeneity of the studies, we performed a pooled analysis after removing each study individually. The results did not change significantly, indicating that the combined results were relatively stable.

### 3.6. Publication bias

**Figure 8:**
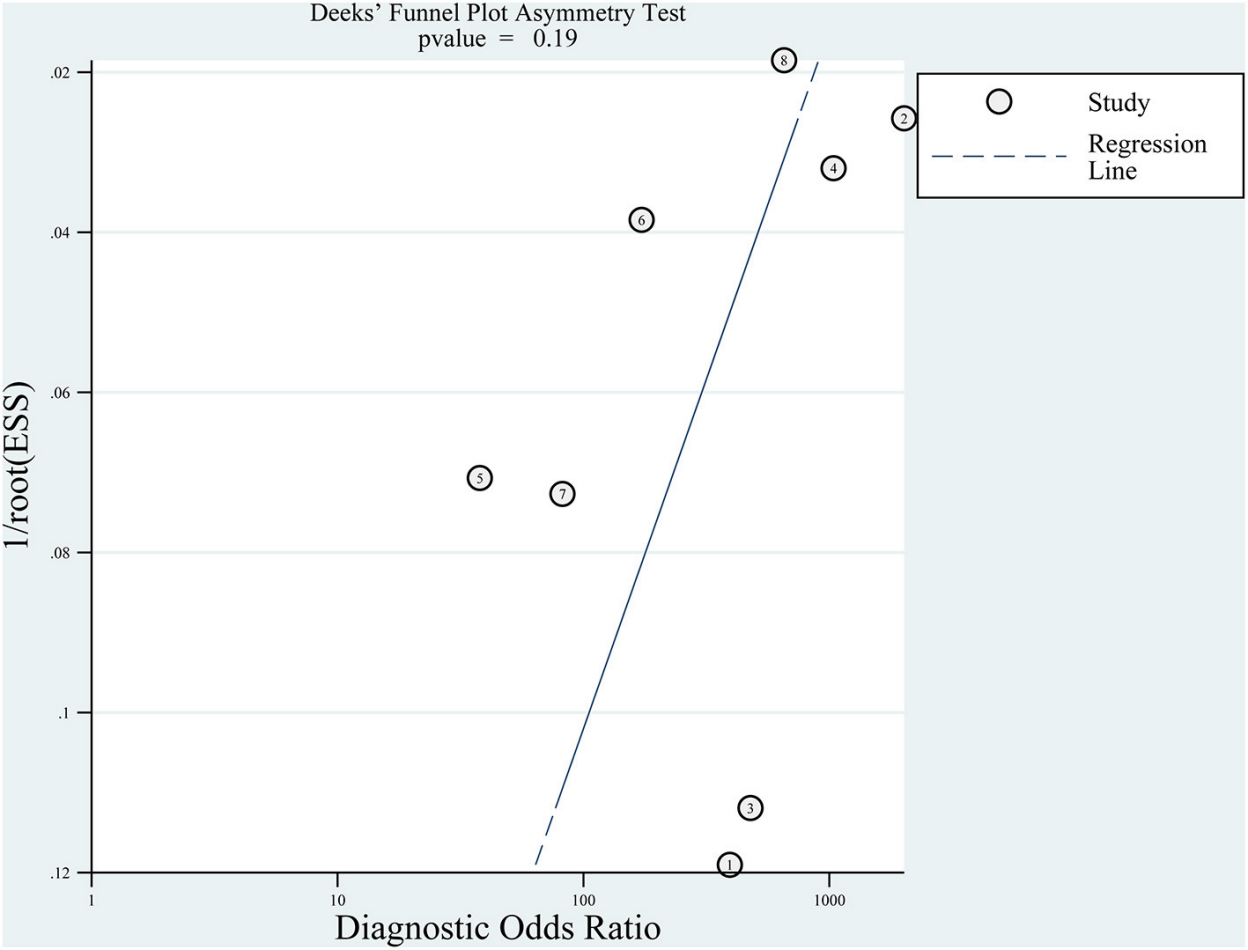
Deeks' funnel plot asymmetry test for publication.

We used the Deeks' funnel plots to evaluate publication bias. As shown in Figure 8, there was no significant publication bias in the included studies (*p* = 0.19). Although the Deeks' test was used, only eight studies were included, and there was still a risk of significant publication bias.

### 3.7. AI vs. endoscopists

From the eight included studies, we extracted five groups of data comparing (AI) with endoscopists (38, 40, 41, 43, 44). An essential condition for inclusion was that the same dataset had to be used for AI vs. endoscopist comparisons.

The study by Qu et al. (39) was excluded because no comparison between AI and endoscopists was found. The study by Luo et al. (42) was excluded because it used a test set containing only gastric sinus images for the comparison of AI with endoscopists. The study by Yang et al. (45) was excluded because it failed to extract the endoscopist's TP, FP, TN, and FN values. Details of the included studies are presented in Table 3.

**Table 3:** AI vs. endoscopist related studies.

**Study**	**Diagnostician**	**TP**	**FP**	**TN**	**FN**
Guimarães et al. (38)	AI	30	5	0	35
	Endoscopist	24	8	6	32
Mu et al. (40)	AI	41	3	1	35
	Endoscopist	37	4	5	34
Lin et al. (41)	AI	357	22	11	706
	Endoscopist	148	118	220	610
Zhao et al. (43)	AI	284	10	54	328
	Endoscopist	212	61	126	277
Xu et al. (44)	AI	14	1	2	7
	Endoscopist	14	3	2	5

We pooled the data of the AI and endoscopists and performed a subgroup analysis. The results were as follows: the sensitivities of AI and endoscopists in diagnosing CAG were 95% (95% CI: 0.91–1.00) and 73% (95% CI: 0.55–0.91) (*p* = 0.1), and the specificities were 96% (95% CI: 0.95–0.98) and 82% (95% CI: 0.78–0.86) (*p* = 0.00), respectively. AI had higher sensitivity and specificity than endoscopists, with no statistically significant difference in sensitivity and a highly significant statistical difference in specificity between the two.

## 4. Discussion

This systematic review and meta-analysis aimed to analyze the accuracy of AI techniques in aiding endoscopic-assisted diagnosis of chronic atrophic gastritis. To our knowledge, this study is the first meta-analysis of the accuracy of endoscopic AI in diagnosing CAG and comparing it with that of endoscopists. A total of eight studies involving 25,216 patients of interest, 84,678 image training set images, and 10,937 test set images/videos were included.

The overall performance of AI in diagnosing CAG was pooled. The pooled sensitivity and specificity were 94% (95% CI: 0.88–0.97, I^2^ = 96.2%) and 96% (95% CI: 0.88–0.98, I^2^ = 98.04%), respectively. The composite AUC and DOR to assess diagnostic accuracy were 98% (95% CI: 0.96–0.99) and 320.19 (95% CI: 128.5–797.84, I^2^ = 100%), respectively. The above data suggest that AI has an excellent diagnostic performance for CAG on endoscopic images or videos. We further compared the performance of AI with that of endoscopists in diagnosing CAG and found that the sensitivity and specificity of AI were significantly higher than those of endoscopists.

There was high heterogeneity among the included studies. Meta-regression analysis was used to determine whether pure normal white light endoscopy or other enhanced endoscopy was used and whether the test set consisted of pictures or videos that did not significantly affect the pooled diagnostic results. The different algorithm types significantly affected the pooled specificity (*p* = 0.03), with the classification algorithm subgroup having a significantly higher specificity than the other algorithm subgroups. However, endoscopists prefer AI to label the lesion site clearly in an image or video. The number of training set images significantly affected the aggregation sensitivity output (*p* = 0.04). The sensitivity of aggregation was significantly higher for the subgroup with more than 7,000 training set pictures than for the subgroup with < 7,000 images. Hence, more training set images may lead to a higher sensitivity. The effect of pathology as the gold standard was highly significant for combined specificity, with only one of the eight studies not using pathology as the gold standard, which could be a potential source of heterogeneity in the pooled results.

This systematic review and meta-analysis had some limitations. (1) Few studies were included, and with eight studies, seven of which were conducted in China, the results may not be representative of the broader population. (2) The pooled results had a high degree of heterogeneity, as not using pathology as the “gold standard,” using different AI algorithms, and using different numbers of training sets are potential sources of heterogeneity. (3) The performance of AI was overestimated. Some studies used test sets with small sample sizes to train the models. Most studies screened training images, which may have caused the AI models to be overfitted. Some studies did not use external test sets or prospective validation sets to test the models, which may have masked the overfitting problem and caused the AI performance to be overestimated.

Despite the excellent performance of AI diagnosis of CAG in endoscopy, there are some pending issues in this area. (1) The performance of AI models cannot be measured uniformly owing to the lack of publicly available datasets. Each study used its own collected dataset for performance evaluation, and different imaging types, image screening processes, and image/video quality can lead to differences in experimental results. (2) Most studies did not include any interference from other diseases. Only two studies identified other diseases (39, 40). Some diseases can seriously impact the mirror diagnosis of CAG, such as erosive gastritis. The performance of AI requires further validation after including the interference from other diseases. (3) Limited replicability of the studies. Most studies included in this systematic review did not make their codes open. This has hindered the validation of their algorithms by other researchers. Code-sharing is essential to repeat the experiment and promote continued progress in the field.

This systematic review and meta-analysis provides a comprehensive overview and quantitative analysis of the current research on AI-assisted diagnosis of CAG and shows that it has good diagnostic performance. Thus, it can be used as an effective auxiliary diagnostic tool in clinical practice. It can provide an accurate diagnosis and reduce the associated time and costs. At the same time, we should also be aware of the limitations of AI models: (1) Limited training data can affect the accuracy and generalization ability of the model. (2) Insufficient diversity of training data can lead to bias in the prediction of the model. (3) The real endoscopic environment is much more complex than the training environment of the model, which may lead to misdiagnosis or missed diagnosis during actual clinical use.

In conclusion, the results of our meta-analysis suggest that AI can provide more accurate diagnostic information on CAG and has high clinical diagnostic value. We hope that our findings will contribute to advance the development and application of AI technology in clinical practice.

## Data availability statement

The datasets presented in this study can be found in online repositories. The names of the repository/repositories and accession number(s) can be found in the article/Supplementary material.

## Author contributions

YS and BL conceived the idea. YS analyzed the data and wrote the manuscript with the support of the other authors. NW, FY, and TT screened the collected data. BL provided suggestions for the project and revised the manuscript. KW validated the statistics and revised the manuscript. All authors discussed the project and read and approved the final manuscript.
